# Comprehensive neuromechanical assessment in stroke patients: reliability and responsiveness of a protocol to measure neural and non-neural wrist properties

**DOI:** 10.1186/s12984-015-0021-9

**Published:** 2015-03-13

**Authors:** Hanneke van der Krogt, Asbjørn Klomp, Jurriaan H de Groot, Erwin de Vlugt, Frans CT van der Helm, Carel GM Meskers, J Hans Arendzen

**Affiliations:** Department of Rehabilitation Medicine, Leiden University Medical Center, Postzone B0-Q, P.O. box 9600, 2300 RC Leiden, the Netherlands; Laboratory for Neuromuscular Control, Faculty of Mechanical, Maritime and Materials Engineering, Delft University of Technology, Mekelweg 2, 2628 CD Delft, the Netherlands; Current address: Department of Rehabilitation Medicine, VU Medical Center, De Boelelaan 1117, 1081 HV Amsterdam, the Netherlands

**Keywords:** Stroke, Biomechanics, Stretch reflex, Wrist, Validation studies

## Abstract

**Background:**

Understanding movement disorder after stroke and providing targeted treatment for post stroke patients requires valid and reliable identification of biomechanical (passive) and neural (active and reflexive) contributors. Aim of this study was to assess test-retest reliability of passive, active and reflexive parameters and to determine clinical responsiveness in a cohort of stroke patients with upper extremity impairments and healthy volunteers.

**Methods:**

Thirty-two community-residing chronic stroke patients with an impairment of an upper limb and fourteen healthy volunteers were assessed with a comprehensive neuromechanical assessment protocol consisting of active and passive tasks and different stretch reflex-eliciting measuring velocities, using a haptic manipulator and surface electromyography of wrist flexor and extensor muscles (Netherlands Trial Registry number NTR1424).

Intraclass correlation coefficients (ICC) and Standard Error of Measurement were calculated to establish relative and absolute test-retest reliability of passive, active and reflexive parameters. Clinical responsiveness was tested with Kruskal Wallis test for differences between groups.

**Results:**

ICC of passive parameters were fair to excellent (0.45 to 0.91). ICC of active parameters were excellent (0.88-0.99). ICC of reflexive parameters were fair to good (0.50-0.74). Only the reflexive loop time of the extensor muscles performed poor (ICC 0.18). Significant differences between chronic stroke patients and healthy volunteers were found in ten out of fourteen parameters.

**Conclusions:**

Passive, active and reflexive parameters can be assessed with high reliability in post-stroke patients. Parameters were responsive to clinical status. The next step is longitudinal measurement of passive, active and reflexive parameters to establish their predictive value for functional outcome after stroke.

**Electronic supplementary material:**

The online version of this article (doi:10.1186/s12984-015-0021-9) contains supplementary material, which is available to authorized users.

## Background

Upper extremity movement disorder is a major contributor to impaired activity and participation levels in post-stroke patients [1,2]. In the acute phase after stroke, paresis is the dominant factor of impairment [3,4]. However, in the chronic phase, the complex interaction between inappropriate neural activation of muscles and secondary biomechanical changes in contractile muscle tissue and passive viscoelastic connective tissue becomes more prominent [3-6]. The dynamical interactions between neural capacity and contractile and connective tissues during daily functioning in patients are poorly understood.

Unraveling movement disorder after stroke into non-neural (passive) and neural (active and reflexive) contributors and assess their separate influence over time, is essential to understand functional recovery after stroke and to aim therapy at the most dominant contributing factor at the most appropriate moment in time [7-10]. Physical examination is currently the most utilized clinical tool for assessment of paresis, inappropriate muscle activity and secondary biomechanical changes [11].

Biomechanical and electrophysiological techniques support standardization of input signals and uniform registration of output signals. A comprehensive neuromechanical assessment should be able to discriminate between non-neural and neural contributors to movement disorder [7-10,12]. Non-neural contributors, i.e. passive tissue properties, should be measured by passive movement at low velocity to minimize background muscle activation [13]. Neural contributors should be measured during active tasks to study voluntary muscle properties and during multiple measurement velocities to study the role of stretch reflexes [3,4,7-10,12]. System Identification and Parameter Estimation techniques assist in separation of neural and non-neural contributors independently of task and condition [14].

Earlier work on measurement of joint neuromechanics [15-19] provided a comprehensive assessment protocol including passive, active and reflexive tests to measure non-neural and neural contributors to movement disorder after stroke [20]. To ensure standardized input signals and registration of output signals, a haptic wrist manipulator [21,22] was combined with surface-EMG measurements.

Clinical implementation of this newly developed protocol required validation. The aim of this study was to assess test-retest reliability and to determine clinical responsiveness in a cohort of stroke patients with upper extremity impairments compared to a cohort of healthy volunteers.

## Methods

### Participants

We identified patients who survived a first ischemic stroke between 1999–2009, and were between 18–80 years at time of stroke, at the outpatient clinics of the Department of Rehabilitation in LUMC and Rijnlands Rehabilitation Center. Inclusion criteria were: a perceived remaining impairment of arm-hand function by the participant, being able to travel to the research laboratory, and being able to sit on a chair and follow instructions for one hour. To establish the perceived impairment of arm-hand function, respondents were asked if they still perceived any impairment of the arm and/or hand. Possible answers were: no impairment, moderate impairment or severe impairment. Patients with moderate to severe perceived impairment were invited for measurements. Exclusion criteria were: limitations of arm-hand function prior to stroke, a history of other neurologic impairments besides stroke. Participants were measured on two occasions within a month, assuming that their clinical status would remain stable. A volunteer sample of healthy volunteers served as a reference group. The study was approved by the Medical Ethical Committee of the LUMC. All participants were compensated for travel expenses.

### Protocol

Measurements were carried out in a laboratory setting at the LUMC. Before the test protocol started, the modified Ashworth Scale (mAS) was measured. Participants were extensively instructed and were given ample opportunity to practice. The protocol consisted of nine tests, with a total duration of approximately 45 minutes.

### Measurement set-up

**Figure 1:**
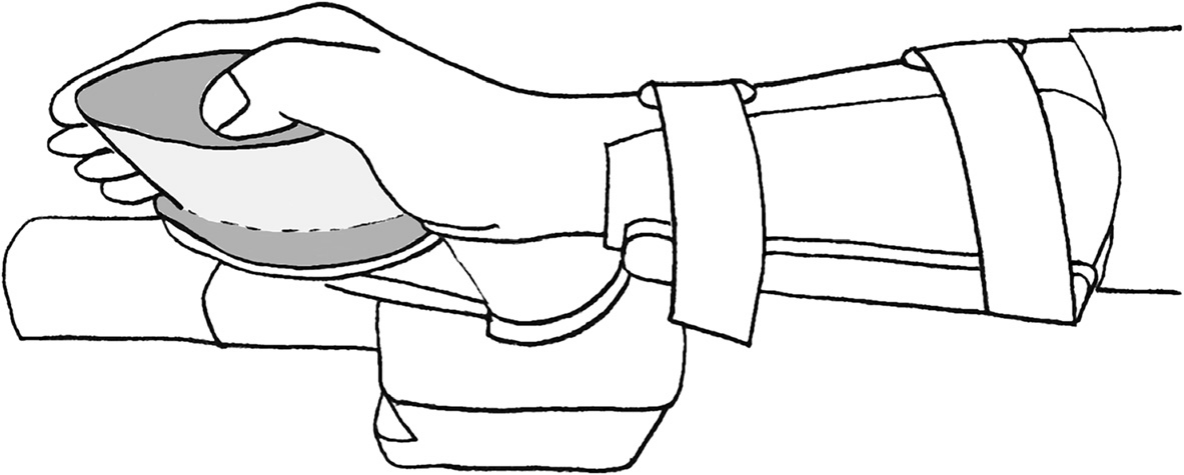
**Illustration of Wristalyzer handle and arm-rest.** For a better view of the hand position, the hand straps are not represented in this illustration.

The measurement set-up [20] consisted of a haptic manipulator (“Wristalyzer”, Moog FCS, the Netherlands) and a surface EMG-system (“Bagnoli”, Delsys Inc., USA). The manipulator delivered precise torque or position perturbations through a vertically positioned servo-motor (Parker SMH100 series), connected to a handle (Meester techniek, the Netherlands). The hand of the participant was fixed to the handle, which had an ellipsoidal shape to prevent finger flexion (Figure 1). The arm was stabilized in an arm rest. The motor axis was aligned with the rotation axis of the wrist joint. Movement of the motor was therefore directly coupled to flexion/extension of the wrist.

### Tasks, conditions and outcome parameters

An overview and more elaborate description of applied Passive, Active and Reflexive tests and their outcome parameters is shown in Table 1. The tests were performed in a fixed order, starting with Passive tests, followed by Active and Reflexive tests respectively [20]. Participants were provided with visual feedback on torque, angle or EMG-level, depending on the test and task instruction.

**Table 1:** **Description of passive, active and reflexive parameters**

**Parameter**		**Description**
**Passive**		
Range of motion passive (degrees)	P_ROM_	Slow passive movement through range of motion, maximum torque is 2 Nm. Range of motion equals the difference between minimal and maximal angle.
Stiffness in rest (Nm rad^−1^)	P_k_	Resistance to passive movement during a slow, position controlled, passive movement through range of motion. Average negative tangent of the hysteresis curve over 0.2 rad around P_RA_.
Rest angle (degrees)	P_RA_	Angle at which the resultant torque during a slow, position controlled, passive movement through range of motion is zero.
**Active**		
Range of motion active (degrees)	A_ROM_	Voluntary movement through range of motion, no resistance from haptic robot. Range of motion equals the difference between minimal and maximal angle.
Maximal voluntary contraction (Nm)	A_MVC_	Maximal torque generated by participant. Fixed position at P_RA_.
Control over joint torque (Nm)	A_CJT_	Ability of participant to achieve steadily increasing target torque. Fixed position at P_RA_.
**Reflexive**		
Threshold angle (degrees)	R_ta_	Angle at which the EMG exceeds 5 times the standard deviation of baseline during a fast, position controlled passive movement through total range of motion.
Reflex loop time (s)	R_lt_	Time from perturbation onset to M_1_-reflex onset. Participant is asked to deliver 10% of maximum EMG-activity during a position controlled movement over 0.14 rad at 3 rad/s.
Reflex contribution to joint resistance (Nms rad^−1^)	R_kv_	Participant is asked to resist fast multisine force pertur-bations. Velocity dependent reflex gain is computed using system identification methods.
Reflex modulation to environment (Nms rad^−1^)	R_m_env_	Participant is asked to resist fast multisine force pertur-bations in a damped environment. Velocity dependent reflex gain in a damped environment is computed.

Adapted from Klomp et al. [20].

Passive tests were performed at low velocity to avoid stretch reflexes, and included a task instruction to “do nothing”. First, a force controlled movement was applied in flexion and extension direction, to establish passive range of motion passive (P_ROM_). Then a position controlled movement was applied, also in both flexion and extension direction. The following outcome parameters were extracted: Stiffness in rest (P_k_) and Rest angle (P_RA_) (Table 1).

Active tests comprised task instructions to “move/push/resist”, i.e. exert a voluntary torque or complete a voluntary movement. This part commenced with an active, maximal movement from flexion to extension and back to establish active range of motion active (A_ROM_). Then, the position of the handle was fixed at the P_RA_ and participants were asked to complete and repeat a maximal voluntary isometric contraction in both flexion and extension direction to establish active maximal voluntary contraction (A_MVC_). Subsequently, participants were asked to match a gradually inclining torque level. This was also performed in both flexion and extension direction and repeated once. The following outcome parameter was extracted: Control over joint torque (A_CJT_) (Table 1).

Reflexive tests were performed at velocities above the assumed stretch reflex threshold, and had either passive or active task instructions. A high velocity, position controlled movement through the P_ROM_ was applied once, in both flexion and extension direction (passive task instruction) to calculate Threshold angle (R_ta_). Short ramp and hold position perturbations were applied 9 times in each direction at random time intervals (active task instruction) to extract Reflexive loop time (R_lt_). A multisine force perturbation was applied for 20 seconds (active task instruction) and repeated three times: once in the same environment and twice in a damped environment. The following outcome parameters were extracted: Reflexive contributions to joint resistance (R_kv_) and Reflex modulation due to environmental changes (R_m_env_) (Table 1).

### Statistical analysis

Data was retrieved and processed with Matlab 2007b (Mathworks, USA) [20]. Calculations and statistics were performed with SPSS Statistics 20 (IBM, USA). Sample size was calculated on the outcome parameter with the expected largest variability: R_kv_. In an earlier study, a standard deviation of 0.17 Nms/rad was found [17], with a mean difference between patients and controls of 0.12 Nms/rad. Based upon α = 0.05 and with a target power of 80%, a sample size of minimally 10 participants was estimated to be required to detect an existing difference between measurements of 0.12 Nms/rad with sufficient power.

Intraclass correlation coefficients (ICC) were calculated using the two-way mixed model for absolute agreement. Values above 0.75 represent excellent reliability, values between 0.4 and 0.75 represent fair to good reliability and values below 0.4 represent poor reliability [23]. As ICC is a relative measure dependent on variance within a group [24], Bland Altman plots were used to illustrate variability and Standard Error of Measurement (SEM) values (Equation 1) and Smallest Detectable Difference (SDD) (Equation 2) were calculated to further substantiate ICC.

1 $$ SEM = SD*\surd \left(1-ICC\right) $$

2 $$ SDD = 1.96\ *\ \surd 2*SEM $$

Normality of distribution was assessed by visual inspection of histograms and equality of variance was tested with Levene’s test. Median, minimum and maximum were calculated per parameter. Levene’s test showed wider variances in the group of chronic stroke patients compared to healthy volunteers, therefore chronic stroke patients were split in two groups according to mAS (mAS = 0 and mAS ≥ 1), a clinimetric observation. This allowed for a more specific description of phenotypes and more equally dispersed values within each group, which was illustrated with box plots. An exploratory comparison was made between parameters of the group of healthy volunteers, the group of chronic patients with mAS = 0 and the group of chronic patients with mAS ≥ 1, using the non-parametric Kruskal-Wallis test. The Kruskal-Wallis test was performed on the average outcome of the two visits per parameter, after testing for systematical differences between the two visits. This comparison was further substantiated by pairwise testing between each of the groups with the non-parametric Wilcoxon Rank Sum test.

## Results

### Descriptive data

We identified and invited 102 post stroke patients. Response rate was 64% (n = 65). Of the responders, 17 patients declined to participate and 16 patients had either no current impairment of arm-hand function or had severely impaired mobility preventing them from travelling to the clinic. Therefore, 32 patients were included in the study. Fourteen healthy volunteers served as a reference group. All healthy volunteers completed the two visits and 28 out of 32 patients completed all visits (87.5%). Reasons for dropping out were: unable to schedule the second visit (n = 2), visit was too tiresome (n = 1), patient was treated with botulinum toxin in period between first and scheduled second visit (n = 1). In chronic stroke patients, the affected hand was dominant in 14 patients (right hand n = 13, left hand n = 1) and non-dominant in 18 patients (right hand n = 2, left hand n = 16). Average age at stroke was 55.2 years. Average time after stroke was 40 months. Descriptive data are presented in Table 2.

**Table 2:** **Descriptive data of the study population**

**Population**	**Healthy volunteers (n = 14)**	**Chronic patients mAS = 0 (n = 21)**	**Chronic patients mAS ≥ 1 (n = 11)**
Age (years) (SD)	49.4 (15.1)	60.4 (13.1)	54.5 (12.7)
Men (n) (%)	9 (64%)	10 (48%)	3 (27%)
Right side dominant (n) (%)	13 (93%)	21 (100%)	8 (73%)
Measured side dominant (n) (%)	14 (100%)	10 (48%)	4 (36%)
Time between measurements (days) (SD)	27 (21)	18 (7)	29 (17)
Time after stroke (months) (SD)	-	30 (27.6)	53 (34.3)
Age at moment of stroke (years) (SD)	-	58 (13.1)	50 (14.5)

Means and standard deviation or percentages for healthy volunteers and chronic stroke patients.

### Reliability

**Figure 2:**
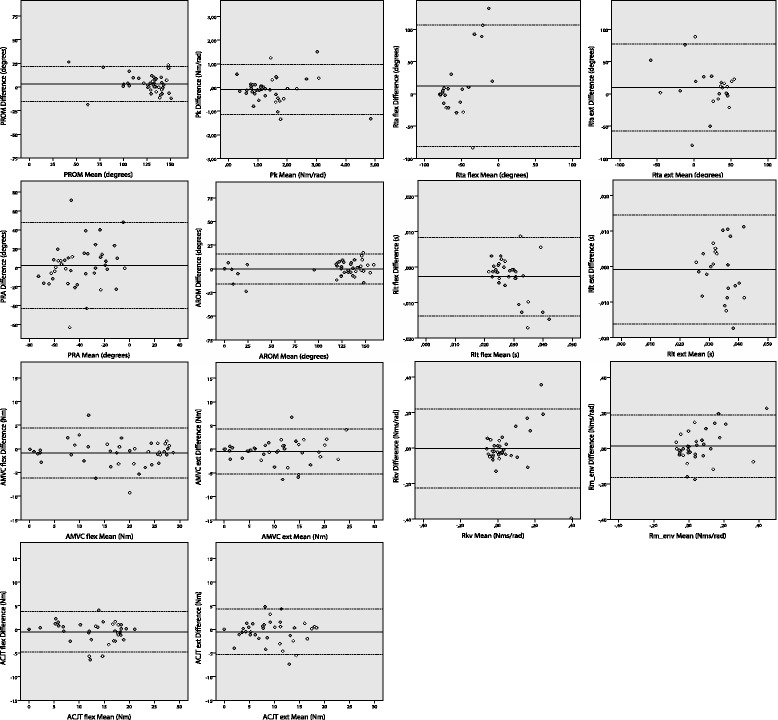
**Bland altman plots for passive, active and reflexive parameters.** P_ROM_: Range of motion passive, P_k_: Stiffness in rest, P_RA_: Rest angle. A_ROM_: Range of motion active, A_MVC_: Maximal voluntary contraction, A_CJT_: Control over joint torque. R_ta_: Threshold angle, R_lt_: Reflexive loop time, R_kv_: Reflexive contributions to joint resistance, R_m_env_: Reflex modulation due to environmental changes. Open circle: Healthy participant. Gray circle: Post-stroke patient. Solid line: mean of the difference between first en second visits. Dotted line: upper and lower limit of 95% confidence interval for difference between first and second visits.

Test-retest reliability for Passive parameters was excellent for P_ROM_ and P_k_ (ICC 0.81 and 0.91 respectively) and fair to good for P_RA_ (ICC 0.45). For Active parameters, test-retest reliability was excellent (ICC 0.88-0.99). For Reflexive parameters, the ICC’s of R_ta_ (flexor and extensor), R_lt_ (flexor), R_kv_ and R_m_env_ were fair to good (ICC 0.50-0.74). ICC for R_lt_ (extensor) was poor (ICC 0.18). ICC’s are summarized in Table 3. Bland Altman plots are shown in Figure 2, depicting the mean of the two measurements (x-axis) compared to the difference between two measurements (y-axis). The values are scattered around the mean difference (solid line), illustrating the absence of a systematic difference or learning effect between the two measurements. In parameters with a lower ICC, the 95% confidence interval of the difference between the measurements is wider, illustrating a larger measurement error. SEM values (Table 3) provide an indication of the dispersion of the measurement errors and SDD are given for future reference (Table 3).

**Table 3:** **Median, minimum and maximum, intraclass correlation coefficients (ICC), standard errors of measurement (SEM) and smallest detectable difference (SDD) for passive, active and reflexive parameters for all participants**

**Parameter**		**All participants median [min; max]**	**ICC**	**SEM**	**SDD**
**Passive**					
P_ROM_ (degrees)		132 [42; 151]	0.91	7	20
P_k_ (Nm rad^−1^)		1.16 [0.29; 4.84]	0.81	0.36	1
P_RA_ (degrees)		−44 [−73; 1]	0.45	14	39
**Active**					
A_ROM_ (degrees)		127 [0; 158]	0.99	5	14
A_MVC_ (Nm)	flexor	17.8 [0.1; 28.7]	0.95	2.1	6
	extensor	10.0 [0.1; 25.4]	0.93	1.8	5
A_CJT_ (Nm)	flexor	12.6 [0.0; 21.1]	0.92	1.9	5
	extensor	7.8 [0.0; 18.4]	0.88	1.9	5
**Reflexive**					
R_ta_ (degrees)	flexor	−68 [−84; 47]	0.67	19*	53*
	extensor	22 [−69; 55]	0.50	26	73
R_lt_ (s)	flexor	0.029 [0.021; 0.045]	0.51	0.0039	0.0109
	extensor	0.035 [0.020; 0.049]	0.18	0.0056*	0.0154*
R_kv_ (Nms rad^−1^)		0.019 [−0.059; 0.395]	0.52	0.0634	0.18
R_m_env_ (Nms rad^−1^)		−0.007 [−0.086; 0.444]	0.74	0.0557	0.15

P_ROM_: Range of motion passive, P_k_: Stiffness in rest, P_RA_: Rest angle. A_ROM_: Range of motion active, A_MVC_: Maximal voluntary contraction, A_CJT_: Control over joint torque. R_ta_: Threshold angle, R_lt_: Reflexive loop time, R_kv_: Reflexive contributions to joint resistance, R_m_env_: Reflex modulation due to environmental changes. *: average of SEM and SDD for R_ta_ flexor and R_lt_ extensor, valid around median of both parameters. Because of heteroscedasticity, SDD and SEM might be smaller towards minimum of parameter and might be larger towards maximum of parameter.

### Responsiveness to clinical status

**Figure 3:**
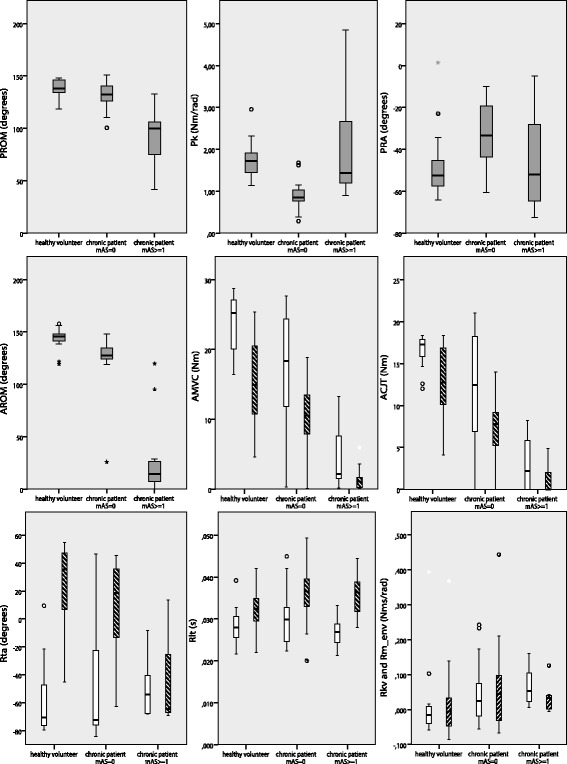
**Box plots for Passive, Active and Reflexive parameters.** Groups divided in healthy volunteers, chronic stroke patients with mAS = 0 and chronic patients with mAS ≥1. P_ROM_: Range of motion passive, P_k_: Stiffness in rest, P_RA_: Rest angle. A_ROM_: Range of motion active, A_MVC_: Maximal voluntary contraction, A_CJT_: Control over joint torque. R_ta_: Threshold angle, R_lt_: Reflexive loop time, R_kv_: Reflexive contributions to joint resistance, R_m_env_: Reflex modulation due to environmental changes. White bars: flexor (and R_kv_ in the lower right panel). Striped bars: extensor (and R_m_env_ in the lower right panel).

An overview of outcomes per group is summarized in Table 4. Dropout rates were similar in both chronic patient groups (mAS = 0: n = 2 and mAS ≥ 1: n = 2). Differences between healthy volunteers, chronic stroke patients with mAS = 0 and chronic stroke patients with mAS ≥ 1 are illustrated by box plots in Figure 3. Corresponding quartiles are given in Additional file 1. In 10 out of 14 parameters, these differences were statistically significant, based on the exploratory Kruskal Wallis test (Table 4). When tested pairwise with the Wilcoxon Rank Sum test (Additional file 2), there were significant differences between healthy volunteers and the mAS = 0 group in the Passive parameters P_k_ and P_RA_; the Active parameters A_ROM_, A_MVC_ (flexor and extensor) and A_CJT_ (flexor and extensor); and the Reflexive parameters R_lt_ (extensor) and R_kv_. Differences between healthy volunteers and the mAS ≥ 1 group showed significance in P_ROM_, all Active parameters, R_ta_ (extensor) and R_kv_. When comparing the mAS = 0 group and mAS≥1 group, there were significant differences in P_ROM_, P_k_ and all Active parameters, but no significant differences in Reflexive parameters.

**Table 4:** **Median, minimum and maximum for healthy volunteers, chronic patients with modified Ashworth score (mAS) = 0 and chronic patients with mAS ≥ 1, and p-value of the Kruskal Wallis test for differences between groups for passive, active and reflexive parameters**

**Parameter**		**Healthy volunteers median [min; max]**	**Chronic patients mAS = 0 median [min; max]**	**Chronic patients mAS ≥ 1 median [min; max]**	**Kruskal Wallis**
**Passive**					
P_ROM_ (degrees)		138 [118; 148]	132 [100; 151]	100 [42; 133]	p < 0.001^#^
P_k_ (Nm rad^−1^)		1.72 [1.13; 2.95]	0.85 [0.29; 1.68]	1.44 [0.90; 4.84]	p < 0.001^#^
P_RA_ (degrees)		−52 [−64; 1]	−33 [−61; −10]	−52 [−73; −5]	p = 0.013^#^
**Active**					
A_ROM_ (degrees)		146 [119; 158]	128 [26; 148]	14 [0; 120]	p < 0.001^#^
A_MVC_ (Nm)	flexor	25.2 [16.4; 28.7]	18.4 [0.3; 27.6]	2.2 [0.1; 13.3]	p < 0.001^#^
	extensor	14.9 [4.6; 25.4]	10.5 [0.1; 18.9]	1.1 [0.1; 6.0]	p < 0.001^#^
A_CJT_ (Nm)	flexor	17.3 [12.0; 18.4]	12.4 [0.0; 21.1]	2.2 [0.0; 8.2]	p < 0.001^#^
	extensor	12.7 [4.1; 18.4]	7.8 [0.0; 14.0]	0.0 [0.0; 4.9]	p < 0.001^#^
**Reflexive**					
R_ta_ (degrees)	flexor	−71 [−80; 9]	−72 [−84; 47]	−54 [−68; −8]	p = 0.221
	extensor	35 [−45; 55]	19 [−63; 46]	−64 [−69; 14]	p = 0.031^#^
R_lt_ (s)	flexor	0.028 [0.022; 0.039]	0.030 [0.022; 0.045]	0.027 [0.021; 0.033]	p = 0.537
	extensor	0.032 [0.022; 0.042]	0.037 [0.020; 0.049]	0.036 [0.028; 0.044]	p = 0.097
R_kv_ (Nms rad^−1^)		−0.015 [−0.059; 0.395]	0.025 [−0.056; 0.242]	0.053 [0.005; 0.160]	p = 0.017^#^
R_m_env_ (Nms rad^−1^)		−0.006 [−0.086; 0.368]	0.044 [−0.068; 0.444]	0.032 [−0.006; 0.126]	p = 0.192

P_ROM_: Range of motion passive, P_k_: Stiffness in rest, P_RA_: Rest angle. A_ROM_: Range of motion active, A_MVC_: Maximal voluntary contraction, A_CJT_: Control over joint torque. R_ta_: Threshold angle, R_lt_: Reflexive loop time, R_kv_: Reflexive contributions to joint resistance, R_m_env_: Reflex modulation due to environmental changes.

^#^: significant difference between groups.

## Discussion

Using a dedicated, comprehensive neuromechanical assessment protocol, Passive and Active parameters could be assessed with excellent reliability in a cohort of stroke patients with upper extremity impairments and healthy volunteers. Repetitive assessment of the Passive parameter P_RA_ and Reflexive parameters had fair to good reliability, except for poor reliability of R_lt_ (extensor). Parameters were responsive to clinical status, i.e. results demonstrated differences between healthy volunteers and chronic stroke patients.

The use of a haptic robot in combination with surface EMG provides standardized application of input signals and registration of output signals. Participants could comfortably tolerate the position in the measurement set-up and the length of the protocol. Previous publication of measurement set-up, protocol and data processing [20] and current assessment of test-retest reliability and clinical responsiveness add to the clinical validity of our method, which is advantageous for prospective implementation of this method in clinical practice.

Relative reliability expressed by ICC’s is both determined by heterogeneity of the study group and the variance on the repeated measurements. In homogenous study groups, relative reliability may drop. For future assessment of longitudinal changes, variability of neuromechanical outcome parameters is unknown and may be dependent on the time of measurement, i.e. low heterogeneity early after stroke when the paresis component prevails and large heterogeneity when secondary biomechanical changes become manifest. We therefore adopted the present cohort-approach to minimize a-priori assumptions on heterogeneity within groups.

Measures of absolute reliability can be used to calculate the SDD, i.e. the difference between measurements that can be attributed to real system changes. For example in A_ROM_ the SDD is 14 degrees, meaning that a change of 14 degrees or over can be attributed to a genuine change in patient characteristics in 95% of cases. SEM values for P_ROM_, P_k_, A_ROM_, A_MVC_ and A_CJT_ were low. These are sensitive parameters for real system changes, indicative for both passive as well as active contributors to observed movement disorders. We therefore recommend these parameters for future assessment of longitudinal neuromechanical changes.

In all Passive, Active and Reflexive parameters except P_k_, P_RA_ and R_lt_ (extensor), differences between healthy volunteers and patients in the mAS ≥ 1 group were more pronounced than differences between healthy volunteers and patients in the mAS = 0 group. However, parameters did not always increase or decrease proportionally between groups, which illustrates the complex and non-linear nature of movement disorder after stroke. In the mAS = 0 group, the paresis component probably plays an important role, while the ability for voluntary motor control is more preserved than in the mAS ≥ 1 group (as can be seen from A_MVC_ and A_CJT_), leading to a lower stiffness (P_k_) in passive structures (i.e. muscle, tendon, ligament). Test-retest results showed good reproducibility, however, the remarkable inter-individual variation in passive and active parameters in the group of chronic patients may represent the different phenotypes in post stroke motor control.

### Strengths and limitations

The perturbations in our protocol may not have been enough to trigger the stretch reflex threshold of the extensor muscles, which are more difficult to trigger [25]. This could have contributed to a lower repeatability in Reflexive parameters R_lt_ (extensor) and R_ta_ (extensor), and a larger SDD than expected for R_kv_. Other contributing factors may be found in a low signal to noise ratio, i.e. absence of inappropriate muscle activity in healthy volunteers and in chronic stroke patients with mAS = 0. Furthermore, variability in Reflexive parameters is known to be present in both healthy volunteers and chronic patients [26-29], even in optimal circumstances. Stretch reflex behavior is more variable than passive tissue properties and voluntary muscle properties [29-31]. Apart from day-to-day variability in stretch reflex behavior [27,29], there is also variability due to level of arousal, audiovisual stimuli and other environmental factors [32,33], as well as conscious down- or up-regulation [34]. These methodological considerations, combined with the unequal variances in subgroups, might account for heteroscedasticity, especially in R_ta_ flexor and R_lt_ extensor. SEM and SDD for these values should be interpreted bearing in mind that SDD and SEM might be smaller towards the minimum of the parameter and might be larger towards the maximum of both parameters.

Although sufficient for the aim of this study, group sizes were small. Current subdivision of clinical phenotypes according to mAS is a fairly rough approximation of clinical status and more participants may be needed for a more elaborate post-hoc analysis. The adopted cohort approach is an estimation of group heterogeneity. The neuromechanical assessment protocol aimed to identify passive, active and reflexive contributors to movement disorder by differences in task and measurement conditions. For example: the protocol was designed to minimize the effects of active and reflexive (neural) contributors during passive (non-neural) tasks and vice versa. However, this might not yet give a completely true reflection of neuromechanical behavior, as system behavior under active task conditions involves a combination of both neural and non-neural contributors. The same goes for passive conditions, where neural components may be present through increased baseline activation [13]. Further development of System Identification and Parameter Estimation techniques might help to zoom in even closer on the specific contributors to neuromechanical behavior.

### Implications for future work

One of the objectives of the EXPLICIT-stroke project [35] will be to combine the neuromechanical approach with extensive clinimetric data. Simultaneously, in this project, longitudinal measurements will be used to provide information on the changes in paresis, the development of secondary biomechanical changes and the increase of inappropriate muscle activity over time. This should provide the necessary data to enhance description of clinical phenotypes by clustering of neuromechanical parameters, and, moreover, to predict functional outcome.

## Conclusions

Passive, Active and Reflexive parameters, representing passive tissue properties, voluntary muscle function and stretch reflex behavior respectively, can be measured in a reliable way. The comprehensive neuromechanical assessment protocol is responsive to clinical status and fulfills the requirements to separately assess non-neural and neural contributors to movement disorder around the wrist after stroke, using biomechanical, electromyographical and system identification techniques [7-10,12]. Therefore, this protocol gives momentum to future work on connecting pathophysiology to functional outcome, which will enable clinicians to substantiate their treatment.

## Additional files

Additional file 1:
**Quartiles of Passive, Active and Reflexive parameters for healthy volunteers, chronic patients with modified Ashworth score (mAS) = 0 and chronic patients with mAS ≥ 1.** P_ROM_: Range of motion passive, P_k_: Stiffness in rest, P_RA_: Rest angle. A_ROM_: Range of motion active, A_MVC_: Maximal voluntary contraction, A_CJT_: Control over joint torque. R_ta_: Threshold angle, R_lt_: Reflexive loop time, R_kv_: Reflexive contributions to joint resistance, R_m_env_: Reflex modulation due to environmental changes. Additional file 2:
**Pairwise comparison with Wilcoxon Rank Sum test between healthy volunteers, chronic patients with mAS = 0 and chronic patients with mAS ≥ 1.** ^#^: significant difference between pair. The Kruskal Wallis test results are repeated from Table 4 for reference. P_ROM_: Range of motion passive, P_k_: Stiffness in rest, P_RA_: Rest angle. A_ROM_: Range of motion active, A_MVC_: Maximal voluntary contraction, A_CJT_: Control over joint torque. R_ta_: Threshold angle, R_lt_: Reflexive loop time, R_kv_: Reflexive contributions to joint resistance, R_m_env_: Reflex modulation due to environmental changes.

## Abbreviations

LUMC: Leiden University Medical Center
mAS: Modified Ashworth Scale
ICC: Intraclass correlation coefficient
SEM: Standard error of measurement
SDD: Smallest detectable difference
EMG: Electromyography
