# Supplementary material for: Comprehensive neuromechanical assessment in stroke patients: reliability and responsiveness of a protocol to measure neural and non-neural wrist properties
Source: J Neuroeng Rehabil. 2015 Mar 13;12:28. doi: 10.1186/s12984-015-0021-9 (PMC4436851; doi:10.1186/s12984-015-0021-9)
Supplement: Additional file 1: — Quartiles of Passive, Active and Reflexive parameters for healthy volunteers, chronic patients with modified Ashworth score (mAS) = 0 and chronic patients with mAS ≥ 1. PROM: Range of motion passive, Pk: Stiffness in rest, PRA: Rest angle. AROM: Range of motion active, AMVC: Maximal voluntary contraction, ACJT: Control over joint torque. Rta: Threshold angle, Rlt: Reflexive loop time, Rkv: Reflexive contributions to joint resistance, Rm_env: Reflex modulation due to environmental changes. [file 12984_2015_21_MOESM1_ESM.docx]

| Parameter |  | 25^th^ percentile | 50^th^ percentile | 75^th^ percentile |
| --- | --- | --- | --- | --- |
| Passive |  |  |  |  |
| P_ROM_  (degrees) | healthy volunteer | 133 | 138 | 146 |
|  | chronic patient  mAS=0 | 126 | 132 | 141 |
|  | chronic patient  mAS>=1 | 75 | 100 | 112 |
| P_k_  (Nm rad^-1^) | healthy volunteer | 1.43 | 1.72 | 2.01 |
|  | chronic patient  mAS=0 | 0.76 | 0.85 | 1.06 |
|  | chronic patient  mAS>=1 | 1.15 | 1.44 | 2.75 |
| P_RA_  (degrees) | healthy volunteer | -58 | -52 | -43 |
|  | chronic patient  mAS=0 | -45 | -33 | -19 |
|  | chronic patient  mAS>=1 | -66 | -52 | -27 |
| Active |  |  |  |  |
| A_ROM_  (degrees) | healthy volunteer | 141 | 146 | 149 |
|  | chronic patient  mAS=0 | 124 | 128 | 136 |
|  | chronic patient  mAS>=1 | 7 | 14 | 45 |
| A_MVC_ flexor  (Nm) | healthy volunteer | 19.5 | 25.2 | 27.2 |
|  | chronic patient  mAS=0 | 11.8 | 18.4 | 25.0 |
|  | chronic patient  mAS>=1 | 1.4 | 2.2 | 7.8 |
| A_MVC_ extensor  (Nm) | healthy volunteer | 10.4 | 14.9 | 20.6 |
|  | chronic patient  mAS=0 | 7.0 | 10.5 | 13.9 |
|  | chronic patient  mAS>=1 | 0.2 | 1.1 | 2.1 |
| A_CJT_ flexor  (Nm) | healthy volunteer | 15.6 | 17.3 | 17.9 |
|  | chronic patient  mAS=0 | 6.6 | 12.4 | 18.3 |
|  | chronic patient  mAS>=1 | 0.0 | 2.2 | 6.1 |
| A_CJT_ extensor  (Nm) | healthy volunteer | 9.9 | 12.7 | 17.1 |
|  | chronic patient  mAS=0 | 4.9 | 7.8 | 10.2 |
|  | chronic patient  mAS>=1 | 0.0 | 0.0 | 2.2 |
| Reflexive |  |  |  |  |
| R_ta_ flexor  (degrees) | healthy volunteer | -77 | -71 | -41 |
|  | chronic patient  mAS=0 | -76 | -72 | -13 |
|  | chronic patient  mAS>=1 | -68 | -54 | -36 |
| R_ta_ extensor  (degrees) | healthy volunteer | 3 | 35 | 47 |
|  | chronic patient  mAS=0 | -16 | 19 | 36 |
|  | chronic patient  mAS>=1 | -69 | -64 |  |
|  |  |  |  |  |
| R_lt_ flexor  (s) | healthy volunteer | 0.025 | 0.028 | 0.031 |
|  | chronic patient  mAS=0 | 0.024 | 0.030 | 0.033 |
|  | chronic patient  mAS>=1 | 0.024 | 0.027 | 0.030 |
| R_lt_ extensor  (s) | healthy volunteer | 0.029 | 0.037 | 0.035 |
|  | chronic patient  mAS=0 | 0.032 | 0.036 | 0.040 |
|  | chronic patient  mAS>=1 | 0.031 | 0.036 | 0.039 |
| R_kv_  (Nms rad^-1^) | healthy volunteer | -0.040 | -0.015 | 0.010 |
|  | chronic patient  mAS=0 | -0.021 | 0.025 | 0,084 |
|  | chronic patient  mAS>=1 | 0.016 | 0.053 | 0.105 |
| R_m_env_  (Nms rad^-1^) | healthy volunteer | -0.053 | -0.006 | 0.033 |
|  | chronic patient  mAS=0 | -0.032 | 0.044 | 0.122 |
|  | chronic patient  mAS>=1 | -0.001 | 0.032 | 0.041 |
|  |  |  |  |  |
