# Supplementary material for: Comprehensive neuromechanical assessment in stroke patients: reliability and responsiveness of a protocol to measure neural and non-neural wrist properties
Source: J Neuroeng Rehabil. 2015 Mar 13;12:28. doi: 10.1186/s12984-015-0021-9 (PMC4436851; doi:10.1186/s12984-015-0021-9)
Supplement: Additional file 2: — Pairwise comparison with Wilcoxon Rank Sum test between healthy volunteers, chronic patients with mAS = 0 and chronic patients with mAS ≥ 1. #: significant difference between pair. The Kruskal Wallis test results are repeated from Table 4 for reference. PROM: Range of motion passive, Pk: Stiffness in rest, PRA: Rest angle. AROM: Range of motion active, AMVC: Maximal voluntary contraction, ACJT: Control over joint torque. Rta: Threshold angle, Rlt: Reflexive loop time, Rkv: Reflexive contributions to joint resistance, Rm_env: Reflex modulation due to environmental changes. [file 12984_2015_21_MOESM2_ESM.docx]

| Parameter |  | Kruskal Wallis | Wilcoxon rank sum test  (between groups) | | |
| --- | --- | --- | --- | --- | --- |
|  |  |  | Healthy volunteers | Healthy volunteers | Chronic patients |
|  |  |  |  |  | mAS=0 |
|  |  |  | vs. | vs. | vs. |
|  |  |  | Chronic patients | Chronic patients | Chronic patients |
|  |  |  | mAS=0 | mAS≥1 | mAS≥1 |
| Passive |  |  |  |  |  |
| P_ROM_ (degrees) |  | p<0.001^#^ | p=0,059 | p<0.001^#^ | p<0.001^#^ |
| P_k_  (Nm rad^-1^) |  | p<0.001^#^ | p<0.001^#^ | p=0.682 | p=0.001^#^ |
| P_RA_ (degrees) |  | p=0.013^#^ | p=0.004^#^ | p=0.725 | p=0.063 |
| Active |  |  |  |  |  |
| A_ROM_ (degrees) |  | p<0.001^#^ | p=0.001^#^ | p<0.001^#^ | p<0.001^#^ |
| A_MVC_ (Nm) | flexor | p<0.001^#^ | p=0.026^#^ | p<0.001^#^ | p<0.001^#^ |
|  | extensor | p<0.001^#^ | p=0.031^#^ | p<0.001^#^ | p<0.001^#^ |
| A_CJT_  (Nm) | flexor | p<0.001# | p=0.086 | p<0.001^#^ | p<0.001^#^ |
|  | extensor | p<0.001^#^ | p=0.002^#^ | p<0.001^#^ | p<0.001^#^ |
| Reflexive |  |  |  |  |  |
| R_ta_ (degrees) | flexor | p=0.221 | p=0.596 | p=0.094 | p=0.202 |
|  | extensor | p=0.031^#^ | p=0.096 | p=0.032^#^ | p=0.057 |
| R_lt_ (s) | flexor | p=0.537 | p=0.711 | p=0.413 | p=0.288 |
|  | extensor | p=0.097 | p=0.041^#^ | p=0.102 | p=0.856 |
| R_kv_ (Nms rad^-1^) |  | p=0.017^#^ | p=0.043^#^ | p=0.006^#^ | p=0.268 |
| R_m_env_ (Nms rad^-1^) |  | p=0.192 | p=0.138 | p=0.076 | p=0.845 |
|  |  |  |  |  |  |
